# Neuropsychological and Socio-Occupational Functioning in Young Psychiatric Outpatients: A Longitudinal Investigation

**DOI:** 10.1371/journal.pone.0058176

**Published:** 2013-03-01

**Authors:** Rico S. C. Lee, Daniel F. Hermens, M. Antoinette Redoblado-Hodge, Sharon L. Naismith, Melanie A. Porter, Manreena Kaur, Django White, Elizabeth M. Scott, Ian B. Hickie

**Affiliations:** 1 Clinical Research Unit, Brain and Mind Research Institute, University of Sydney, Sydney, Australia; 2 Department of Psychology, Macquarie University, Sydney, Australia; 3 Child Development Unit, The Children’s Hospital at Westmead, Sydney, Australia; The Nathan Kline Institute, United States of America

## Abstract

**Background:**

Clinical symptoms and neuropsychological deficits are longitudinally associated with functional outcome in chronic psychiatric cohorts. The current study extended these findings to young and early-course psychiatric outpatients, with the aim of identifying cognitive markers that predict later socio-occupational functioning.

**Methods:**

At baseline, 183 young psychiatric outpatients were assessed. Ninety-three returned for follow-up (M = 21.6 years old; SD = 4.5) with an average re-assessment interval of 21.6 months (SD = 7.0), and primary diagnoses of major depressive disorder (*n* = 34), bipolar disorder (*n* = 29), or psychosis (*n* = 30). The primary outcome measure was cross-validated with various other functional measures and structural equation modelling was used to map out the interrelationships between predictors and later functional outcome.

**Results:**

Good socio-occupational functioning at follow-up was associated with better quality of life, less disability, current employment and being in a romantic relationship. The final structural equation model explained 47.5% of the variability in functional outcome at follow-up, with baseline neuropsychological functioning (a composite of memory, working memory and attentional switching) the best independent predictor of later functional outcome. Notably, depressive and negative symptoms were only associated with functioning cross-sectionally. Diagnosis at follow-up was not associated with functional outcome.

**Conclusions:**

Neuropsychological functioning was the single best predictor of later socio-occupational outcome among young psychiatric outpatients. Therefore, framing psychiatric disorders along a neuropsychological continuum is likely to be more useful in predicting functional trajectory than traditional symptom-based classification systems. The current findings also have implications for early intervention utilising cognitive remediation approaches.

## Introduction

Traditionally in mental health, there has been a strong emphasis on defining recovery in terms of symptom resolution. However, there is increasing recognition that defining remission solely on the basis of clinical symptoms is inadequate. Frequently, these ‘remitted’ patients with diagnoses of major depression [1], bipolar disorder [2] or psychosis [3] remain functionally impaired. Zimmerman et al. [1] demonstrated that half of patients with major depression who were classified as remitted based on rating scale criteria did not consider themselves to be *in remission*. Critically, these individuals were significantly more impaired in their work performance and social relationships. It is likely that subsyndromal symptoms, particularly depression [2] and negative symptoms [4], contribute to these ‘residual’ functional deficits across the major psychiatric disorders. Of significance, cognitive vulnerabilities may *additionally* contribute to persisting disability given evidence that neuropsychological dysfunction predicts later socio-occupational functioning, over and above symptom levels, as observed in depression [5], bipolar disorder [6], and schizophrenia [7].

While in chronic cohorts psychotic disorders are associated with more pronounced neuropsychological and psychosocial disability than affective disorders [8], neuropsychological performance appears to predict functional outcome irrespective of diagnosis [8]. Accordingly, neuropsychological deficits have been proposed as endophenotypes that cut across traditional diagnostic boundaries [9]. This idea of intermediate traits spanning the major psychiatric disorders is not new, and is buttressed by findings of neurometabolic [10], neuroanatomical [11], and neurophysiological [12] factors common to major depression, bipolar disorder and psychosis. The overarching premise is that affective and psychotic disorders may exist along a continuum [13], and exploring shared dimensional features is likely to be more informative prognostically, and for the understanding of pathophysiology (e.g. for identifying suseptibility genes) [14], than categorising individuals according to their idiosyncratic symptomatology [15]. Indeed, within-disorder heterogeneity is not uncommon in the current diagnostic classification systems. Furthermore, separating patients based on syndromes is often confounded by varying illness trajectories, as well as the finding that diagnoses can be relatively unstable, particularly in the early stages of psychiatric illness [16].

To date, there is strong evidence that cognitive dysfunction is shared across early-course major depression [17], bipolar disorder [18], and schizophrenia [19]. Consistent with studies in well-established cohorts, neuropsychological deficits appear longitudinally predictive of functional outcome over and above symptomatology in early psychosis [20] and possibly, in first-episode mania [21]. To our knowledge, such relationships have not been explored in first-episode major depression. Identification of cognitive markers with predictive value may ultimately help to direct early intervention strategies. For example, some deficits may be indicative of underlying structural and functional brain changes, which in turn may be amenable to novel pharmacotherapies, or cognitive remediation, with the latter already showing some promise as an early intervention strategy [22].

The aim of the current study was to determine whether neuropsychological functioning longitudinally predicts socio-occupational functioning over and above symptomatology in a young mixed psychiatric sample with major depression, bipolar disorder or psychosis. We hypothesised that neuropsychological functioning, as well as depressive and negative symptoms, would contribute to later functional outcome. Structural equation modelling techniques were used to estimate the interrelationships among clinical variables more accurately than past studies utilising multivariate regression approaches [23].

## Materials and Methods

### Ethics Statement

The current study was approved by the University of Sydney Human Research Ethics Committee. After complete description of the study to the subjects at each assessment time-point, those aged 16 years and above gave written informed consent to participate, whereas for those under the age of 16 years both the participant and their legal guardian gave written informed consent in accordance with Human Research Ethics Committee guidelines and Australian law.

### Subjects

Participants were recruited from specialised tertiary referral services (‘Treatment Centre’) for the assessment and early intervention of mental health problems in young people (Youth Mental Health Clinic, YMHC, at the Brain & Mind Research Institute, BMRI; and *headspace*, Campbelltown, Sydney, Australia; Inner West Area Health Service First Episode Psychosis Intervention Services) [24]. Inclusion criteria were restricted to persons aged 12 to 35 years seeking professional help primarily for a depressive (unipolar or bipolar) and/or psychotic syndrome. Subjects were excluded if they were diagnosed with a neurological condition, had insufficient English-language skills, or intellectual disability (premorbid IQ<70). Patients were assessed by a psychiatrist according to DSM-IV TR [American Psychiatric Association, 25] criteria at two time points (and diagnoses were subsequently confirmed through case-note review), and underwent a comprehensive clinical and neuropsychological assessment (including self-report questionnaires) by clinical neuropsychologists or trained research psychologists at baseline. A repeat clinical assessment (with self-report questionnaires) was also conducted at follow-up.

### Measures

The BMRI Structured Interview for Neurobiological Studies was conducted to gather key demographic and clinical information [26]. Symptoms were rated using the expanded version of the Brief Psychiatric Rating Scale (BPRS-E) [27]. Subscores were calculated based on a previously derived component structure (depressive, positive, negative and manic symptoms) [28].

Premorbid intellectual functioning (premorbid IQ) was estimated using the Wechsler Test of Adult Reading (WTAR) [29] or Wide Ranging Achievement Test – fourth edition (WRAT-4; for participants younger than 16 years) [30]. Various neuropsychological tests were also administered, including Trail Making Test – Part A and B (TMT-A and TMT-B) [31], Logical Memory II Percentage Retention [LM-Ret; participants younger than 16 years (*n* = 7) were excluded from this variable as there was no equivalent delayed retention index] [32], Rey Auditory Verbal Learning Test 20-Minute Percentage Retention (RAVLT-Ret) [33], Rapid Visual Processing Hits (RVP-Hits), Spatial Span length (SSP), Paired Associates Learning adjusted errors (PAL), and Intra-/Extradimensional Shift total errors (IED) from the Cambridge Neuropsychological Test Automated Battery (CANTAB) [34], and Letter (FAS) and Category (animals) Fluency [35]. Neuropsychological scores were standardised into z-scores adjusting for demographic variables based on internal (LM-Ret, CANTAB) [32] and external normative samples (TMT-A & TMT-B, RAVLT-Ret, Letter and Category Fluency) [36]–[38].

Various socio-occupational measures were administered. The Social and Occupational Functioning Scale (SOFAS) rated by the treating psychiatrist and research psychologist [39] was the primary functional outcome measure used. Additional patient-rated information relating to work and study, financial and living situation, and relationship status, and structured questionnaires, including total scores from the World Health Organization Quality of Life BREF version (WHOQOL-BREF) [40] and the Disability Assessment Scale II (WHODAS-II) [41], were included to cross-validate the clinician-rated SOFAS.

### Data Analysis

Statistical analyses were conducted using Statistical Package for the Social Sciences Version 20.0 (SPSS 20.0) and Analysis of Moment Structures Version 20.0 (AMOS 20.0). Participants were initially dichotomised into two groups, as adapted from similar methodology [42], based on their follow-up SOFAS scores. Scores greater than or equal to 61 were classed as ‘good outcome’, whereas a lesser score was categorised as ‘poor outcome’. This dichotomy was chosen since it was more meaningful to examine outcome groups anchored to qualitative descriptors (i.e. ‘generally functioning well or better’ compared with ‘moderate impairment or worse’) than using correlation coefficients based on a continuous SOFAS score. In other words, dichotomising the SOFAS score means that the sample characteristics are immediately interpretable in reference to whether patients are functioning well or poorly. Analyses of Variance (ANOVAs; Welch’s correction where homogeneity of variance was violated according to Levene’s test) and chi-squared tests were conducted to cross-validate the clinician-rated SOFAS score with other objective and subjective functional indices. ANOVAs were also conducted on baseline variables to explore potential factors associated with follow-up SOFAS outcome. Neuropsychological indices that differed according to outcome group were reduced to latent variables by confirmatory factor analysis.

Since structural equation modelling is particularly sensitive to the full range of statistical variability, continuous SOFAS scores were used in this procedure. A two-step approach to structural equation modelling was adopted [43]. First, the measurement model (latent neuropsychological functioning) was tested for model fit. Second, we tested the structural model (the relationships between measured variables) using a model-trimming approach through an iterative process in which non-significant paths with the smallest contribution were sequentially eliminated from a saturated model (where all variables were allowed to correlate) until a best fitting model was ascertained to explain the relationships between baseline symptoms and follow-up functioning. All baseline cross-sectional relationships were treated as correlational due to uncertainty of directionality. The model was improved through the inclusion of follow-up symptoms and compared to the original model using nested-models. Latent baseline neuropsychological functioning was then entered into the model to determine whether it explained functional outcome over and above other predictors, again using nested-models. Overall model fit was determined using the *χ^2^* statistic, Bentler comparative fit index (CFI) [44], Bentler-Bonnett nonnormed fit index (NFI) [45], and Root Mean Square Error of Approximation (RMSEA) [46] with 90% confidence interval. A good-fitting model was indicated by a non-significant *χ^2^* test (indicating non-significant difference between the covariance matrix of the data and the model), a CFI and NFI of greater than 0.90 (indicating that the current model was superior to a null model where all paths are constrained to zero), and a RMSEA of less than 0.05 with an upper confidence interval bound of less than 0.08 (indicating that the error of approximation of the model compared with the data was acceptable). Nested-models were compared using Δ*χ^2^* with significant changes representative of better model fit. In the case of non-significant Δ*χ^2^*, the model with greater degrees of freedom (i.e. more parsimonious) was considered the superior model [47].

## Results

In total, 183 patients diagnosed with major depressive disorder (*n* = 66), bipolar disorder (*n* = 52) and psychosis (*n* = 65) were assessed at baseline. Of these, 93 patients (48 males), aged 12 to 34 years of age (M = 21.6, SD = 4.5), returned for follow-up (34 major depressive disorder, 29 bipolar disorder, and 30 psychosis). Psychiatric comorbidities were common, with additional diagnoses of social anxiety disorder (*n* = 15), generalised anxiety disorder (*n* = 8), obsessive-compulsive disorder (*n* = 8), borderline personality disorder (*n* = 4), attention-deficit/hyperactivity disorder (*n* = 3), and narcissistic personality disorder (*n* = 1). Age of onset of psychiatric symptoms was relatively young (M = 16.2, SD = 4.7), although this measure reflected the emergence of psychiatric symptoms and not the onset of full-threshold diagnoses according to DSM-IV TR.

Patients did not return for follow-up for various reasons, including those who were disinterested in participation (*n* = 23), not contactable or had transportation difficulties (*n* = 54), too busy with full-time work or study (*n* = 8), too ill (*n* = 4), or had deceased (*n* = 1). Compared with those who returned for follow-up, those who did not were not significantly different in terms of diagnoses, age, age of onset, years of education, depressive, positive, negative or manic symptoms, or on any neuropsychological measure (all *p*>0.14). However, returning patients had significantly higher premorbid IQ [104 vs. 100; F(1, 177) = 6.73, *p* = .01], although this no longer remained significant once outliers (*n* = 6) with premorbid IQ less than 80 or greater than 120 were excluded (*p*>.10). Nonetheless, even by including patients with outlying premorbid IQs the difference is unlikely to be clinically significant given the discrepancy of 4 IQ points (equates to a standard deviation difference of 0.27). Thus, these patients were not excluded from subsequent structural equation modelling analyses.

### Cross-validating SOFAS at Follow-Up

At follow-up, a high inter-rater agreement was obtained between treating psychiatrists and research psychologists on the SOFAS measure (intraclass correlation coefficient = 0.778, p<.000) [48], [49]. Using our qualitatively-defined dichotomy, 53 patients met criteria for ‘good outcome’, whereas 40 patients were classified as ‘poor outcome’ (see Table 1). Good outcome patients reported greater overall quality of life [F(1,62.2) = 6.65, *p*<.05], less disability [F(1,67.0) = 5.67, *p*<.05], were more often employed [56.6% vs. 15.0%, *χ^2^*(1) = 16.63, *p*<.001], in a romantic relationship [43.4% vs. 17.5%, *χ^2^*(1) = 7.00, *p*<.01], and less likely to be on a disability support pension [13.2% vs. 45.0%, *χ^2^*(1) = 11.72, *p*<.001]. The mean follow-up interval was 21.6 months (SD = 7.0; range 6–48 months), and this neither differed between outcome groups nor correlated with follow-up SOFAS score (*p*>.05).

**Table 1:** Characteristics of poor and good functional outcome groups at follow-up.

Variables	Poor Outcome (*n* = 40)		Good Outcome (*n* = 53)		Statistics		
*Clinical*	*M*	*SD*	*M*	*SD*	*F*	*p-value*	*Effect Size* c
BPRS Depression	13.39	6.0	12.16	3.7	*1.40*	*ns*	*0.25*
BPRS Positive	11.14	5.2	10.45	3.7	*0.53*	*ns*	*0.16*
BPRS Negative	8.44	3.3	7.16	2.9	*3.67*	*ns*	*0.41*
BPRS Mania	9.44	3.1	9.82	4.0	*0.23*	*ns*	*0.10*
*Functional*							
SOFAS	51.99	7.1	71.38	6.9	*173.72* a	*0.000*	*2.75*
WHOQOL-BREF	47.57	11.8	53.63	8.5	*6.65* a	*0.012*	*0.60*
WHODAS-II	36.70	19.0	26.69	15.0	*5.67* a	*0.020*	*0.59*
	***N***	***%***	***N***	***%***	***χ^2^***	***p-value***	***Effect Size*** d
Employed	6	15.0	30	56.6	*16.63*	*0.000*	*0.42*
Student	17	42.5	31	58.5	*2.33*	*ns*	*0.16*
On a Disability Support Pension	18	45.0	7	13.2	*11.72*	*0.001*	*0.36*
Has a Romantic Partner	7	17.5	23	43.4	*7.00*	*0.008*	*0.27*
Living with Parents or Relative(s)	29	72.5	32	60.4	*1.48*	*ns*	*0.13*
Living with Flatmate(s)	5	12.5	7	13.2	*0.01*	*ns*	*0.01*
Living with Partner	2	5.0	8	15.1	*2.42*	*ns* b	*0.16*
Living Alone	4	10.0	6	11.3	*0.04*	*ns* b	*0.02*

BPRS = Brief Psychiatric Rating Scale. SOFAS = Social and Occupational Functioning Assessment Scale. WHOQOL-BREF = World Health Organization Quality of Life (BREF) Scale. WHODAS-II = World Health Organization Disability Assessment Schedule II.

a Welch’s statistic correcting for homogeneity of variance violation.

b Fisher’s exact test correcting for cells with *n*<5.

c Hedges’ *g.*

d Cramer’s *V.*

### Characteristics of Outcome Groups

Age, age of onset, gender, years of education, and premorbid IQ did not differ between outcome groups (*p*>.05; see Table 2). Patients with good outcome were functioning better at baseline according to the SOFAS [F(1,91) = 18.70, *p*<.001]. There were more bipolar patients in the good outcome group (*n* = 23, 43.4%) relative to the poor outcome group [*n* = 6, 15.0%; *χ^2^*(1) = 8.57, *p*<.01], although by contrast manic symptoms were more elevated in the poor outcome group at baseline [F(1,52.3) = 6.74, *p*<.05]. Further inspection of the data suggested that this anomaly was driven by the bipolar patients in the poor outcome group who rated significantly higher on the BPRS mania scale than those in the good outcome group [F(1,27) = 15.68, *p*<.001]. There were more psychosis patients in the poor outcome group (*n* = 18, 45.0%) than the good outcome group [*n* = 12, 22.6%; *χ^2^*(1) = 5.22, *p*<.05]. There were neither significant differences between the proportion of major depression patients in the good (*n* = 18, 34%) compared with the poor outcome group [*n* = 16, 40%; *χ^2^*(1) = 0.36, *p* = .55], nor between the number of patients with comorbidities between the good (*n* = 20, 40%) and poor outcome groups [*n* = 19, 35.8%; *χ^2^*(1) = 1.88, *p* = .17]. Treatment centre was not associated with outcome (*p*>.05). Most patients were medicated (*n* = 71, 76.3%), although on average more patients in the poor outcome group were taking medication [87.5% vs. 67.0%; *χ^2^*(1) = 4.84, *p*<.05]. Specifically, more patients were treated with antipsychotics in the poor outcome group, consistent with its higher proportion of psychosis patients [54.0% vs. 43.4%, *χ^2^*(1) = 5.33, *p*<.05]. There were no differences between outcome groups on antidepressant or anticonvulsant/mood stabiliser usage (*p*>.44). At baseline, patients with poor outcome performed significant worse on tests of working memory [SSP; F(1,91) = 10.44, p<.005], verbal memory retention [LM-Ret; F(1,91) = 9.53, *p*<.005], visual memory [PAL; F(1,51.3) = 10.50, *p*<.005], and attentional switching [TMT-B; F(1,65.5) = 6.75, *p*<.05].

**Table 2:** Characteristics of poor and good outcome groups at baseline.

Variables	Poor Outcome (*n* = 40)		Good Outcome (*n* = 53)		Statistics		
*Demographic*	*M*	*SD*	*M*	*SD*	*F*	*p-value*	*Effect Size* c
Age (years)	21.75	4.3	21.42	4.7	*0.13*	*ns*	*0.07*
Age of Onset (years)	16.87	4.6	15.77	4.7	*1.19*	*ns*	*0.23*
Education (years)	12.43	2.6	12.75	2.5	*0.39*	*ns*	*0.12*
Premorbid IQ	103.85	10.2	104.40	8.9	*0.08*	*ns*	*0.06*
*Clinical*							
BPRS Depression	14.23	5.2	13.06	5.8	*1.02*	*ns*	*0.21*
BPRS Positive	11.77	4.8	11.08	3.7	*0.61*	*ns*	*0.16*
BPRS Negative	8.38	3.0	7.36	2.9	*2.73*	*ns*	*0.34*
BPRS Mania	11.72	5.3	9.30	2.7	*6.74* a	*0.012*	*0.60*
*Neuropsychological*							
TMT-A	−0.24	1.1	−0.10	1.3	*0.29*	*ns*	*0.11*
TMT-B	−0.93	1.7	−0.12	1.2	*6.75* a	*0.012*	*0.56*
LM-Ret	−0.65	1.2	0.08	1.0	*9.53*	*0.003*	*0.66*
RAVLT-Ret	−0.70	1.5	−0.21	1.4	*2.66*	*ns*	*0.34*
RVP-Hits	−1.05	1.5	−0.75	1.3	*0.93*	*ns*	*0.21*
SSP	−0.52	1.2	0.24	1.0	*10.44*	*0.002*	*0.69*
PAL	−0.88	1.7	0.10	0.9	*10.50* a	*0.002*	*0.75*
IED	−0.43	1.4	−0.10	1.1	*0.47*	*ns*	*0.26*
Letter Fluency	−0.45	1.1	−0.12	1.0	*2.26*	*ns*	*0.31*
Category Fluency	−0.04	1.4	0.35	1.0	*2.45*	*ns*	*0.33*
*Functional*							
SOFAS	54.89	9.4	64.33	10.6	*18.70*	*0.000*	*0.93*
WHOQOL-BREF	45.52	11.4	48.58	8.1	*1.90* a	*ns*	*0.31*
WHODAS-II	41.34	16.8	39.81	18.1	*0.13*	*ns*	*0.09*
*Treatment Centre*	*N*	*%*	*N*	*%*	*χ^2^*	*p-value*	*Effect Size* d
BMRI or Headspace	30	75.0	42	79.2	*0.24*	*ns*	*0.05*
FEP Team	5	12.5	4	7.5	*0.64*	*ns* b	*0.08*
External Clinician	5	12.5	7	13.2	*0.01*	*ns*	*0.01*
*Medication-Use*							
Medicated	35	87.5	36	67.9	*4.84*	*0.028*	*0.23*
Antidepressant-Use	19	47.5	21	39.6	*0.58*	*ns*	*0.08*
Antipsychotic-Use	27	54.0	23	43.4	*5.33*	*0.021*	*0.24*
Anticonvulsant-Use	4	10.0	5	9.4	*0.01*	*ns* b	*0.01*

BPRS = Brief Psychiatric Rating Scale. TMT-A = Trail Making Test – Part A. RVP-Hits = Rapid Visual Processing Hits. SSP = Spatial Span length. LM-Ret = Logical Memory percentage retention. RAVLT-Ret = Rey Auditory Verbal Learning Test percentage retention. PAL = Paired Associates Learning adjusted errors. TMT-B = Trail Making Test- Part B. IED = Intra−/Extradimensional Set Shift Task total errors. SOFAS = Social and Occupational Functioning Assessment Scale. BMRI = Brain & Mind Research Institute. FEP Team = Inner West Area Health Service First Episode Psychosis Intervention Services.

a Welch’s statistic correcting for homogeneity of variance violation.

b Fisher’s exact test correcting for cells with *n*<5.

c Hedges’ *g.*

d Cramer’s *V.*

### Structural Equation Modelling

The four significant neuropsychological measures identified in the current study were best explained by one latent factor (all other factors had eigenvalues <0.6), which accounted for 60% of the variance in neuropsychological performance among the four measures (SSP, LM-Ret, PAL, TMT-B). Model fit indices were good (*χ^2^* = .67, *df* = 2, *p* = .72, CFI = 1.00, NFI = 0.99, RMSEA = 0.00, 90% CI = 0.00–0.15).

**Figure 1:**
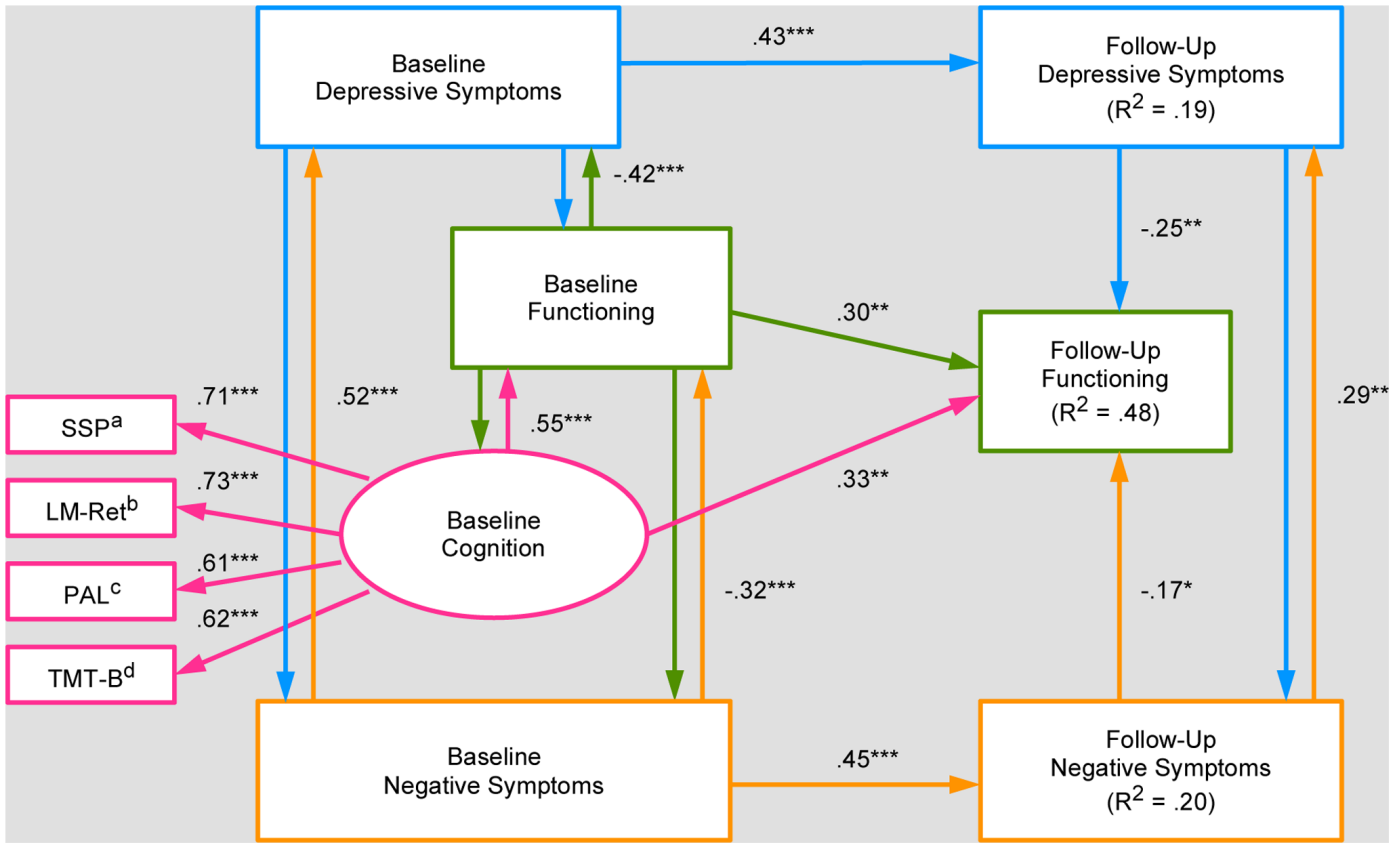
Final structural equation model. *Note:* Rectangles denote measured variables. Ovals denote latent variables. Single-headed arrows represent regression paths. Two single-headed arrows pointing in opposite directions represent correlations. Residual error terms for all endogenous variables were omitted for ease of viewing. SSP = Spatial Span length. LM-Ret = Logical Memory percentage retention. PAL = Paired Associates Learning adjusted errors. TMT-B = Trail Making Test – Part B. Depressive and Negative Symptoms were measured by subscales from the expanded Brief Psychiatric Rating Scale, and Functioning was measured by the Social and Occupational Functioning Assessment Scale. ^a^R^2^ = .50. ^b^R^2^ = .54. ^c^R^2^ = .37. ^d^R^2^ = .39. *p≤.05. **p≤.01. ***p≤.001.

Entering only baseline SOFAS and symptoms into the structural model did not yield any models with uniformly adequate fit indices. The best possible model included depression and negative symptoms (mania did not predict SOFAS outcome despite being different between good and poor outcome groups). This model was somewhat inadequate and paths from symptoms to follow-up functioning, as measured by the SOFAS, were both non-significant (*χ^2^* = 37.33, *df* = 31, *p* = .20, CFI = .97, NFI = .86, RMSEA = 0.05, 90% CI = 0.00–0.10). Modifying the model by including follow-up symptoms as predictors (which were in turn predicted by baseline symptoms) yielded a good fitting model with adequate fit indices (*χ^2^* = 23.68, *df* = 29, *p* = .74, CFI = 1.00, NFI = 0.91, RMSEA = 0.00, 90% CI = 0.00–0.06). This was a significant improvement over the previous model and was thus adopted (Δ*χ^2^* = −13.65, Δ*df* = −2, *p*<.001). As the paths from baseline symptoms to follow-up functioning were still not statistically significant, we tested whether these paths were necessary. The subsequent model, removing direct paths from baseline symptoms to follow-up functioning, was an adequate fit and all paths were significant (*χ^2^* = 27.32, *df* = 31, *p* = .66, CFI = 1.00, NFI = 0.90, RMSEA = 0.00, 90% CI = 0.00–0.07). In addition, it was not significantly different from the previous model (Δ*χ^2^* = 3.64, Δ*df* = 2, *p* = .162) and, thus, it was adopted because it was more parsimonious. Finally, the neuropsychological latent variable was entered into the model as a predictor of follow-up functioning. This model was a good fit with all paths statistically significant (*χ^2^* = 19.00, *df* = 30, *p* = .94, CFI = 1.00, NFI = 0.93, RMSEA = 0.00, 90% CI = 0.00–0.02), fitting better than the previous model (Δ*χ^2^* = −8.32, Δ*df* = −1, *p*<.005) and, thus, was adopted as the final model since the significant increase in model fit justified the reduction in parsimony [47]. The final model (Figure 1) explained 47.5% of follow-up functioning, with the latent baseline neuropsychological variable being the strongest predictor, accounting for 10.9% of the variance.

Since proportions of diagnoses differed between outcome groups when initially dichotomising the SOFAS scores, we decided to test whether being diagnosed with bipolar disorder or psychosis (the two diagnoses that were differentially represented) might have better accounted for the outcomes seen at follow-up. The results revealed that diagnoses did not improve model fit and, to the contrary, made the model less parsimonious (Δ*χ^2^* = −1.85, Δ*df* = −2, *p* = .40). In addition, neither the paths from bipolar disorder nor psychosis to follow-up functioning were significant (*r* = .07 for both predictors, *p*>.40), while baseline neuropsychological functioning remained the best predictor. Therefore, the final model in Figure 1 was retained.

## Discussion

In young psychiatric outpatients relatively early in their illness, moderate or greater functional impairment was associated with poorer quality of life, greater disability, unemployment and single relationship-status. Functional outcome was most strongly predicted by baseline neuropsychological functioning, controlling for initial level of functioning and concurrent symptoms. Baseline verbal and visuospatial memory, spatial working memory, and attentional switching were instrumental to socio-occupational functioning 6 to 48 months later, consistent with findings from more chronic cohorts, which have identified baseline memory [6], [7], [50] and executive functioning [5]–[7] to be predictive of later functioning across our diagnostic groups. Neurobiologically, this may reflect underlying frontotemporal changes, which underpin later functional outcome early in the course of affective and psychotic illness. In contrast, baseline neuropsychological functioning was not associated with symptomatology at baseline or follow-up, in keeping with the literature [21]. Thus, memory and executive functioning are likely independent of clinical state in early-course psychiatric illness, underscoring their potential as endophenotypes.

In contrast, baseline depressive and negative symptoms did not directly predict later functional outcome. These symptoms were, however, associated with functional outcome cross-sectionally, in line with previous findings [51]. Therefore, symptoms and functioning do not appear to be causally linked, but instead may be outcomes of a common pathophysiology. Similarly, the utility of diagnosis in predicting functional outcome was questioned by the current findings. Diagnosis was unrelated to level of functioning over and above the effects of cognition and clinical symptoms, implying that categorising patients based on traditional classification systems is unlikely to be informative in regards to functional trajectory. Taken together, these results strongly suggest that a traditional, or sole, focus on symptom factors is inadequate in characterising prognosis and recovery. On the other hand, the longer-term outcome of young patients presenting to mental health services with a range of disorders is best predicted by their initial level of neuropsychological functioning.

The strong cross-sectional relationship currently found between neuropsychological and socio-occupational functioning contradicts previous reports in more chronic patient populations. For instance, Simonsen et al. [8] found that most neuropsychological measures were not singly predictive of functional outcome in a group of chronic bipolar disorder and schizophrenia patients in their mid-thirties. Moreover, overall neuropsychological and socio-occupational functioning were only modestly correlated. This discrepancy with the current data suggests that the influence of neuropsychological functioning on later functional outcome may be greater in the early stages of illness. That is, over time the impact of neuropsychological deficits on functioning may be diminished as the chronicity of disability is spontaneously maintained. Although this is speculative, it highlights the potential importance of early intervention, since interventions aimed at reducing disability may be particularly effective within this initial, critical period from psychiatric onset [52].

A major strength of the current study was its longitudinal design, providing stronger, directional evidence of the predictive utility of neuropsychological functioning over traditional cross-sectional approaches. Structural equation modelling also permitted a more sophisticated representation of how clinical symptoms, cognition and socio-occupational functioning are inter-related over time. In addition, the use of a naturalistic sample of patients suffering from a range of severe mental illnesses, and frequently with other psychiatric comorbidities, strengthens the ecological validity of the current findings.

### Limitations and Future Directions

A number of limitations must be acknowledged. The current study was restricted by its modest sample size. As such, we were unable to explore whether the relationships between cognition and functional outcome differed according to traditional diagnostic categories using multiple-group modelling procedures [53]. Future studies need to directly test whether diagnosis *moderates* the currently observed effects, as previously demonstrated cross-sectionally in more chronically ill samples [4]. Moreover, studies should include mediators of the relationship between neuropsychological and socio-occupational functioning, which have been identified in cross-sectional studies of chronic schizophrenia and bipolar disorder patients. Indeed, there is evidence suggesting that social competence [54] and social cognition [55] mediate this relationship. Separately, the suggestion that memory and executive functioning may be potential endophenotypes of major psychiatric morbidity needs to be verified in future studies including cohorts of unaffected family members and healthy comparison individuals.

### Summary

The current study was the first longitudinal study utilising structural equation modelling to demonstrate a strong link between neuropsychological and later socio-occupational functioning in young and early-course psychiatric outpatients. Memory and executive dysfunction were linked to later disability, highlighting the potential of reducing longer-term disability through early neuroprotective strategies targeting frontal and temporal brain regions. Thus, psychiatric illness may be better viewed along a neuropsychological continuum, with those on the impaired end more likely to follow a less favourable functional trajectory. A traditional and sole focus on symptoms or syndromes is unlikely to be useful in predicting functional recovery. Compared with more chronic stages, neuropsychological functioning may be a more robust predictor of later functioning in the early stages of psychiatric disease, suggesting that cognitive remediation strategies may be most efficacious in effecting functional change in this early ‘critical period’.
